# Effectiveness of fentanyl buccal soluble film in cancer patients with inadequate breakthrough pain control

**DOI:** 10.1186/s12904-024-01483-7

**Published:** 2024-06-14

**Authors:** Yi-Hao Chiang, Ching-Ting Lien, Wen-Hao Su, Tsung-Yu Yen, Yu-Jen Chen, Yuen-Liang Lai, Ken-Hong Lim, Kun-Yao Dai, Hsin-Pei Chung, Chia-Yen Hung, Yi-Shing Leu

**Affiliations:** 1 Division of Hematology and Oncology, Department of Internal Medicine, MacKay Memorial Hospital, Taipei, Taiwan; 2 Department of Medicine, MacKay Medical College, New Taipei City, Taiwan; 3 Laboratory of Good Clinical Research Center, Department of Medical Research, MacKay Memorial Hospital, New Taipei City, Taiwan; 4 Department of Nursing, MacKay Memorial Hospital, Taipei, Taiwan; 5 Hospice and Palliative Care Center, MacKay Memorial Hospital, Taipei, Taiwan; 6 Department of Radiation Oncology, MacKay Memorial Hospital, Taipei, Taiwan; 7 Department of Death Care Service, Nursing and Management, MacKay Junior College of Medicine, Taipei, Taiwan; 8 Chest Division, Department of Internal Medicine, MacKay Memorial Hospital, Taipei, Taiwan; 9 Department of Otolaryngology-Head Neck Surgery, MacKay Memorial Hospital, No. 45, Minsheng Rd., Tamsui District, New Taipei City, 25160 Taiwan; 10 Cancer Center, MacKay Memorial Hospital, Taipei, Taiwan

**Keywords:** Fentanyl buccal soluble film, FBSF, Breakthrough cancer pain, Around-the‐clock, Rapid-onset opioids, Proportional

## Abstract

**Background:**

Clinical evidence for the rapidity and effectiveness of fentanyl buccal soluble film (FBSF) in reducing pain intensity of breakthrough cancer pain (BTcP) remains inadequate. This study aimed to evaluate the efficacy of FBSF proportional to the around-the‐clock (ATC) opioid regimens in rapidly relieving the intensity of BTcP episodes by determining the percentage of patients requiring further dose titration.

**Methods:**

The study procedure included a dose-finding period followed by a 14-day observation period. Pain intensity was recorded with a Numeric Rating Scale (NRS) at onset and 5, 10, 15, and 30 min after FBSF self-administration. Meaningful pain relief was defined as the final NRS score ≤ 3. Satisfaction survey was conducted for each patient after treatment using the Global Satisfaction Scale.

**Results:**

A total of 63 BTcP episodes occurred in 30 cancer patients. Only one patient required rescue medication at first BTcP episode and then achieved meaningful pain relief after titrating FBSF by 200 µg. Most BTcP episodes relieved within 10 min. Of 63 BTcP episodes, 30 (47.6%), 46 (73.0%), and 53 (84.1%) relieved within 5, 10, and 15 min after FBSF administration. Only grade 1/2 adverse events were reported, including somnolence, malaise, and dizziness. Of the 63 BTcP episodes, 82.6% were rated as excellent/good satisfaction with FBSF.

**Conclusion:**

FBSF can be administrated “on demand” by cancer patients at the onset of BTcP, providing rapid analgesia by achieving meaningful pain relief within 10 min.

**Trial registration:**

This study was retrospectively registered 24 December, 2021 at Clinicaltrial.gov (NCT05209906): https://clinicaltrials.gov/study/NCT05209906.

**Supplementary Information:**

The online version contains supplementary material available at 10.1186/s12904-024-01483-7.

## Introduction

Breakthrough cancer pain (BTcP) caused by tumor itself and/or relevant treatment is a common symptom in cancer patients [1]. Unlike persistent background pain that is stable and manageable with long-acting opioid products, most BTcP is an unpredictable exacerbation of severe pain characterized by short duration and rapid onset [2]. Although the peak pain intensity of most BTcP episodes occurs within minutes and lasts 30–60 min, inadequate relief for BTcP is known to be associated with worse outcomes and poorer health-related quality of life [3, 4]. While management of BTcP remains challenging, the intermittent natural of breakthrough pain flares further pose difficulties in treatment effect interpretations. The prevalence of BTcP varies widely in many literatures, ranging from 20 to 80%, depending on patient characteristics, hospital department or unit, stage of disease, and doses used for background analgesia [5–8]. Many patients are still not satisfied with BTcP, especially oral morphine [9, 10]. Among the existing drug for the treatment of BTcP, oral mucosal absorption of fentanyl is currently the fastest and most effective strategy for relieving BTcP [11].

Given the poor analgesic efficacy of morphine for unpredictable BTcP, rapid-onset opioids (ROOs) are recently considered to be the most suitable medication for “on-demand” treatment of BTcP in cancer patients [12]. Numerous clinical trials and meta-analysis have demonstrated the superior efficacy of transmucosal fentanyl formulations over opioids in counteracting BTcP [13–16]. Fentanyl buccal soluble film (FBSF), as one of the fentanyl formulations, is an approved drug in Taiwan (Painkyl®) and Europe (Breakyl®), which can effectively provide transmucosal delivery of fentanyl for rapid relief of BTcP [17]. The technology uses a dual-layer polymer film comprising a mucoadhesive layer containing the active drug and an inactive layer that hinders the diffusion of drug into the oral cavity. The mucoadhesive layer adheres to a moist mucosal membrane within seconds. FBSF starts to dissolve within minutes and fully dissolved within 15–30 min after application, without requiring any effort from the patient, as it only needs a minimal amount of saliva to dissolve once adhered. Previous studies have indicated that when delivered through this system, approximately 50% of the fentanyl dose undergoes transmucosal absorption, resulting in an absolute bioavailability of around 71% [18]. The direct relationship between the surface area of the dose unit and the dose of fentanyl combined with the mucosa contact time results in consistent plasma concentrations when equivalent doses are delivered by single or multiple dosage units [19, 20]. Since high-intensity BTcP in cancer patients often requires high doses of analgesics, titration from low doses is often ineffective and time-consuming. Therefore, some studies suggest a dose proportionality strategy for fentanyl, as stepwise titration for effective pain relief is too slow in clinical practice, especially in advanced cancer patients in need of high-dose analgesia [21–23].

Several studies have demonstrated the dose proportionality of fentanyl released and absorbed following FBSF administration and its efficacy and safety in relieving BTcP with minimal adverse events [19, 21, 22, 24]. Although the corresponding effective doses of FBSF has been established based on the around-the‐clock (ATC) opioid regimens in oral morphine equivalence [23], it remains unclear how many cancer patients still require further dose titration to effectively and safely relieve BTcP and patient satisfaction with FBSF. Therefore, this prospective study aimed to evaluate whether dose-proportional FBSF could effectively and safely relieve BTcP episodes in cancer patients. The design of the conversion table (Supplementary Table 1) is grounded in clinical practice experiences, where an oral BTcP medication is typically administered at doses equivalent to 1/6 of the ATC dose. This is derived from a previously published article [20]. In addition, this study recorded and analyzed the time series of changes in pain intensity for each BTcP episode at short time intervals after FBSF administration. This is the questionnaire-based study conducted to evaluate the analgesic effect of FBSF on meaningful pain relief in BTcP at the 5, 10, 15, and 30 min, as well as 30 min after the prescription of the FBSF. In addition, the doses of ATC and FBSF in this study can be adjusted according to the patient’s condition. The findings of this study would provide a reference for the efficacy and safety by dose proportionality of FBSF in relieving BTcP in cancer patients.

## Materials and methods

### Study design

This study was a prospective single-arm observational study designed to evaluate the efficacy and safety of proportional doses of FBSF in rapidly relieving the intensity of BTcP episodes in cancer patients at Mackay Memorial Hospital-Tamsui Branch. The study protocol was approved by the Institutional Review Board (19MMHIS032e) at Mackay Memorial Hospital and conducted in accordance with the Declaration of Helsinki. The study procedure consisted of a screening period, a dose-finding period of approximately 7 days, and an observation period of 14 days [19].

### Participants

The study was conducted between February 2019 and June 2022. Written informed consent was obtained from all eligible patients. This study was retrospectively registered on ClinicalTrials.gov (NCT05209906 ||https://clinicaltrials.gov/; December 24, 2021). The inclusion criteria were as follows: (1) patients aged 20–80 years; (2) patients received oral strong opioid analgesics for at least 1 week, and the dose should be equivalent to 60–360 mg/day of oral morphine or 25–150 µg/hour of transdermal fentanyl; (3) at least partial relief of breakthrough pain by use of opioid therapy; (4) patients are able to self-administer the study medication correctly or have an available adult caregiver to administer the study medication correctly; and (5) patients are willing and able to complete patient questionnaire. Subjects with following conditions were excluded from this study: (1) rapidly escalating pain (e.g., regularly more than 3 breakthrough pain episodes per day) that are hard to be controlled by analgesics; (2) history of hypersensitivity or intolerance to fentanyl (3) cardiopulmonary disease that may increase the risk of respiratory depression as judged by the doctor; (4) psychiatric/cognitive or neurological impairment; (5) severe (grade 4) mucositis or stomatitis; (6) abnormal oral mucosa that would prevent drug absorption; (7) a history of alcohol or drug abuse; (8) the use of other investigational drug or rapid-onset opioids within 4 weeks prior to enrollment in this study; and (9) pregnant or breastfeeding women.

### Data collection: dose-finding period

Patients in the dose-finding period received a proportional dose of FBSF (Painkyl®, TTY Biopharm), calculated based on their ATC opioid doses for background cancer pain, as described previously and recorded the current pain intensity before and 5, 10, 15 and 30 min after the use of FBSF [22]. The initial dose of FBSF given to the patient was based on their ATC dose of analgesics (Supplementary Table 1). Assessment of background pain intensity was performed at study entry, along with patients’ background information. Background dose of ATC opioids was converted to oral morphine equivalent daily dose and divided into six categories: 60, 120, 180, 240, 300, and 360 mg/day, which correspond to FBSF doses of 200, 400, 600, 800, 1000 and 1200 µg/time, respectively. If the converted oral morphine dose falls between two categories, the lower dose level was used to calculate the initial FBSF dose. In this study, meaningful pain relief was defined as a final Numeric Rating Scale (NRS) score ≤ 3, and the effective dose of FBSF was defined as the dose that achieved meaningful pain relief within 30 min of FBSF administration without intolerable side effects in two consecutive BTcP episodes entry observation period. Rescue medication is permitted when the BTcP episode cannot be adequately relieved after 30 min of self-administration of FBSF. If the pain intensity of a BTcP episode is not effectively relieved, the dose of FBSF will be titrated by 200 µg in subsequent BTcP episodes until pain relief is achieved (NRS score ≤ 3).

### Data collection: observation period

During the 14-day observation period, self-administered FBSF for analgesia at each BTcP episode and other relevant data on pain management were recorded, including drug name and dose received before and after the onset of BTcP episode.

### Intervention: endpoints and assessments

The primary objective of this study was to evaluate the efficacy of dose-proportional FBSF in rapidly relieving the intensity of BTcP episodes in cancer patients. The secondary objectives included evaluation of efficacy and safety of dose-proportional FBSF. The primary endpoint was the percentage of patients requiring further dose titration. Secondary endpoints included the percentage of change in pain intensity after the use of FBSF analgesics, patients’ satisfaction with FBSF used for BTcP as well as adverse events induced by FBSF.

Pain intensity of the patient was self-assessed using the NRS at the onset of the BTcP and 5-, 10-, 15-, and 30-min following the start of FBSF administration [19]. The score of NRS ranges from 0 (no pain) to 10 (the worst pain as the patient can imagine). The NRS was accompanied with an expressive scale to facilitate easy and accurate reporting of pain scores. The patients were instructed to choose a number that best corresponded with their level of pain. Pain intensity of the patient was further classified into three categories, as follows: 0–3 (mild intensity), 4–7 (moderate intensity), and 8–10 (high intensity). Satisfaction survey with FBSF analgesics was performed for each patient using a 5-point scale (poor, fair, good, very good, and excellent), derived from the Treatment Satisfaction Questionnaire for Medication (TSQM; [23]). The Pain and Satisfaction Survey Questionnaire used in this study is included in the Supplementary data. For safety assessment, the incidence of Adverse Events (AEs) and Serious Adverse Events (SAEs) was graded according to Common Terminology Criteria for Adverse Events (CTCAE) [25] and documented during the study period. The severity and relationship of each AE and SAE were also recorded.

### Statistical analysis

Full analysis set (FAS) contained subjects who received at least 1 dose of FBSF without major protocol violation. Per-protocol (PP) population included subjects who completed study dose-finding period. The safety population was defined as subjects who were exposed to at least 1 dose of FBSF and were available for follow-up safety information. All statistical analyses were performed using SAS software, version 9.4 (SAS Institute, Inc.; Cary, NC). The study planned to enroll a total of 30 subjects, which was based on a previous study [22] and clinical feasibility. Descriptive data are expressed as mean with standard derivation (SD) or as numbers with percentage. The mean and standard error of mean (SEM) are used to present the variability in changes in NRS scores following the administration of FBSF.

## Results

### Patients flow

**Fig. 1:**
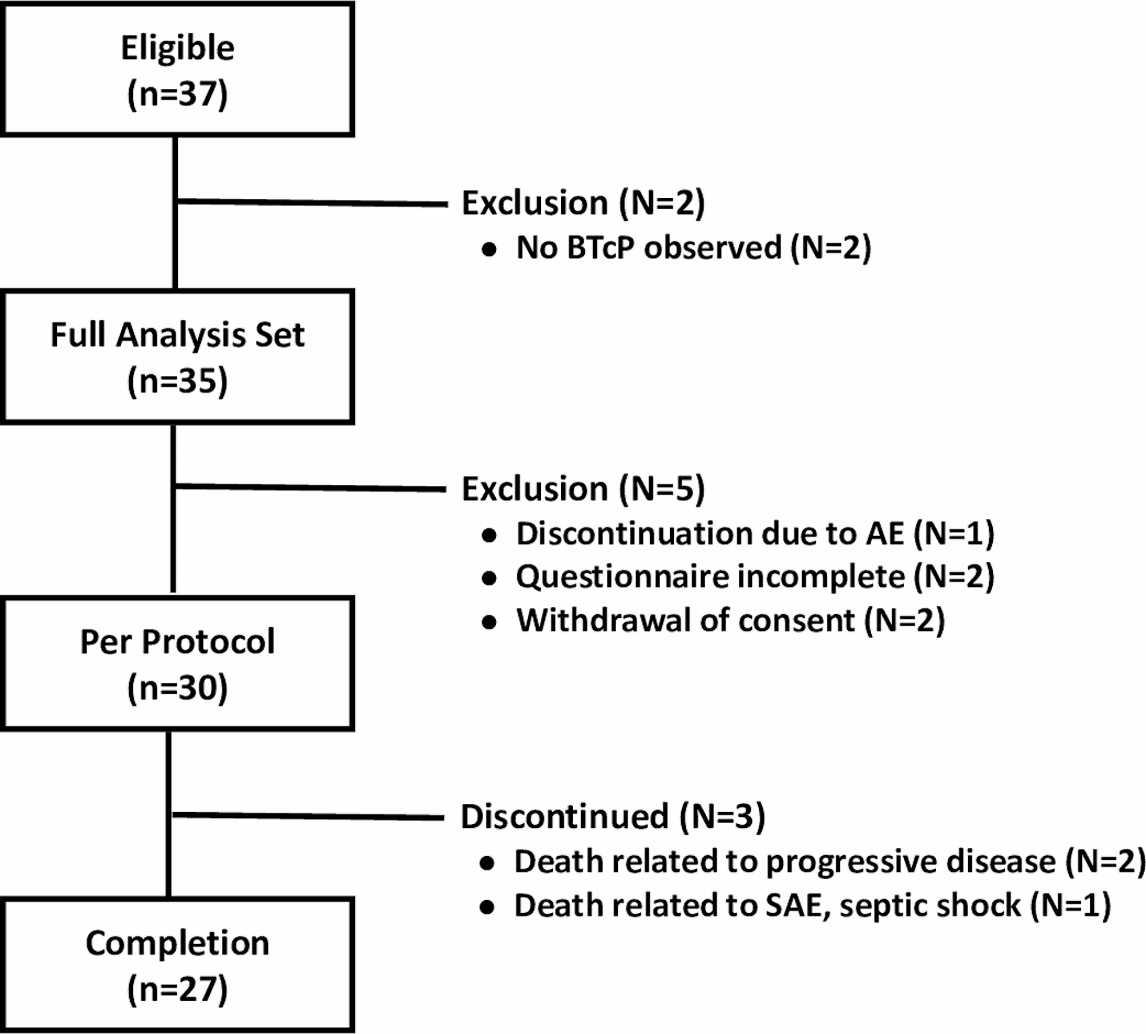
STROBE diagram. The Full Analysis Set was defined as patients who received at least one dose of the study drug. The Per Protocol Set was defined as patients who had correctly received two doses of FBSF to achieve meaningful pain relief. The Completion was defined as patients completed a 14-day observation period and a self-administered questionnaire to assess satisfaction with FBSF. Abbreviation: BTcP, breakthrough cancer pain; AE, adverse event; SAE, serious adverse event

Figure 1 shows the flow chart of the patients in this prospective study. Of the 37 eligible patients enrolled in this study, two patients were excluded because no breakthrough pain was observed after enrollment. The Per Protocol Set included 30 patients, excluding one who discontinued due to AE, two who did not complete the questionnaire, and two who withdrew the consent. For patients in the Per Protocol Set, subjects self-assessed BTcP twice to confirm effective FBSF dose for BTcP and entered a 14-day observation period. Finally, 27 of 30 patients completed a 14-day observation period with effective doses of FBSF for BTcP (Fig. 1, STROBE diagram).

Table 1 summarizes the patient characteristics of the 30 patients. The mean age was 59.1 ± 7.9 years, and nearly two-third of patients were male (63.3%). Among them, there were 36.7% of gastrointestinal cancer, 23.3% of head and neck cancer, 16.7% of lung cancer, 16.7% of genitourinary or gynecological cancer, and 6.7% of breast cancer. Most patients were diagnosed with stage IV (73.3%). In these cancer patients, the background cancer pain was observed in the abdomen (26.7%), limbs (26.7%), and head and neck (20.0%), as well as non-specific location of pain (26.7). The intensity of background cancer pain in most patients was mild (83.3%). All the BTcP occurred in the same location as background pain (100%).

**Table 1:** Baseline characteristics of participants (*N* = 30)

Characteristics		*N*	%
Age, years (mean ± SD)		59.1 ± 7.9	
BMI, kg/m^2^ (mean ± SD)		22.9 ± 4.5	
Gender			
	Male	19	63.3
	Female	11	36.7
Cancer type			
	Gastrointestinal cancer	11	36.7
	Head and Neck cancer	7	23.3
	Lung cancer	5	16.7
	Genitourinary or Gynecological cancer	5	16.7
	Breast cancer	2	6.7
Cancer Stage*			
	I	0	0.0
	II	3	10.0
	III	2	6.7
	IV	22	73.3
Site of background pain			
	Abdomen	8	26.7
	Limbs	8	26.7
	Head and Neck	6	20.0
	Non-specific location	8	26.7
Intensity of background pain^†^			
	Mild (score 0 − 3)	25	83.3
	Moderate (score 4 − 6)	5	16.7
	High (score 7 − 10)	0	0.0
Intensity of BTcP episode^†^			
	Mild (score 0 − 3)	1	3.3
	Moderate (score 4 − 6)	16	53.3
	High (score 7 − 10)	13	43.3

* Missing data on tumor stage information in 3 patients

† Pain intensity was evaluated using the Numeric Rating Scale

Abbreviations: BMI, body mass index; SD, standard deviations; BTcP, breakthrough cancer pain

Head & Neck cancer = hypopharyngeal x2 + oropharyngeal x1 + tongue x1 + right tongue base x1 + gum x1 + lower gum x1; GI cancer = colon x4 + esophageal x3 + gastric x1 + liver x1 + pancreatic x1 + Cholangiocarcinoma x1; GYN/GU cancer = ovarian x1 + cervical x1 + prostate with bone metastasis x1 + bladder x1 + urothelial x1

### Analgesic efficacy of FBSF for BTcP episodes in cancer patients

Most of the background pain were mild intensity (83.3%). The high, moderate, and mild intensity of BTcP accounted for 13 (43.3%), 16 (53.3%), and 1 (3.3%) patient, respectively. The starting FBSF dose of each patient was calculated based on the ATC opioid doses for background pain, as described previously [22]. In this study, patients undergoing treatment received oral morphine at ATC doses ranging from 60 to 119 mg, 120 to 179 mg, 180 to 239 mg, and 240 mg or more. The distribution of patients across these dosage categories was as follows: 18 (60.0%), 8 (26.7%), 3 (10.0%), and 1 (3.3%), respectively (Supplementary Table 1). During the dose-finding period, 21 (70%), 5 (16.7%), 3 (10.0%), and 1 (3.3%) patients were assigned to self-administer 200, 400, 600, and 800 µg of FBSF for BTcP, respectively.

**Fig. 2:**
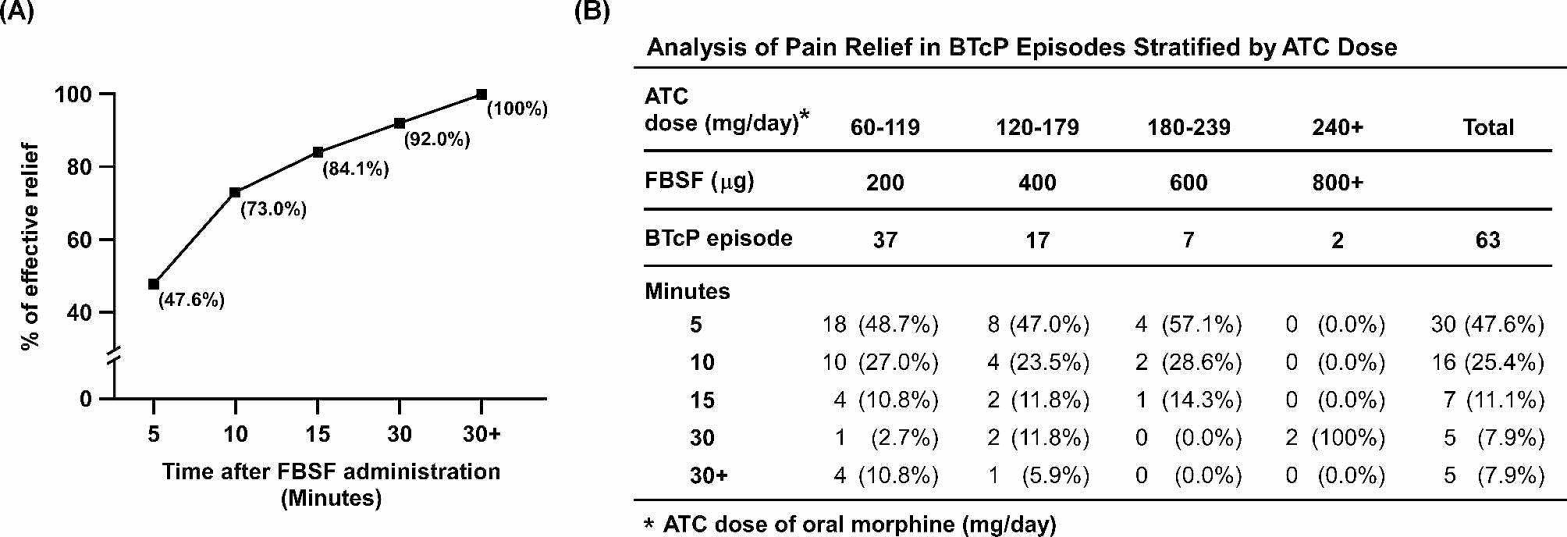
Relief status of BTcP episodes by FBSF during the observation period. **(A)** Cumulative percentage of BTcP episodes effectively relieved by FBSF over time. Numbers in brackets indicate the number of BTcP episodes that were effectively relieved at the indicated time point. **(B)** Stratified analysis of BTcP episodes that effectively relieved by FBSF according to the doses of ATC morphine. Pain intensity of each BTcP episodes was assessed using the NRS at indicated time point. Abbreviation: ATC, around-the-clock; BTcP, breakthrough cancer pain; FBSF, fentanyl buccal soluble film; NRS, Numeric Rating Scale

During the dose-finding period, a total of 63 episodes of BTcP were reported by 30 patients, with an average of 2.1 episodes per patient. For the primary endpoint, only one patient (3.3%) required rescue medication and titration during the first BTcP episode. In this study, meaningful pain relief was defined as a final Numeric Rating Scale (NRS) score ≤ 3, and the effective dose of FBSF was defined as the dose achieving meaningful pain relief without intolerable side effects. Afterwards, the dose of FBSF was titrated from 200 µg to 400 µg and meaningful pain relief was achieved. Figure 2A shows the percentage of BTcP episodes at each time point that achieved meaningful pain relief. Of the 63 BTcP episodes, 47.6%, 73.0%, 84.1%, and 92.0% of BTcP episodes were meaningfully relieved within 5, 10, 15, and 30 min after administration of FBSF. Similar results were observed when categorizing the 63 BTcP episodes based on the dose of oral morphine they received (ATC doses of 60–119 mg, 120–179 mg, 180–239 mg, and 240 mg or more). At 10 min, the number of patients with BTcP effectively relieved (NRS score ≤ 3) were 28 (75.7%), 12 (70.5%), 6 (85.7%) and 0 (0%), respectively (Fig. 2B).

**Fig. 3:**
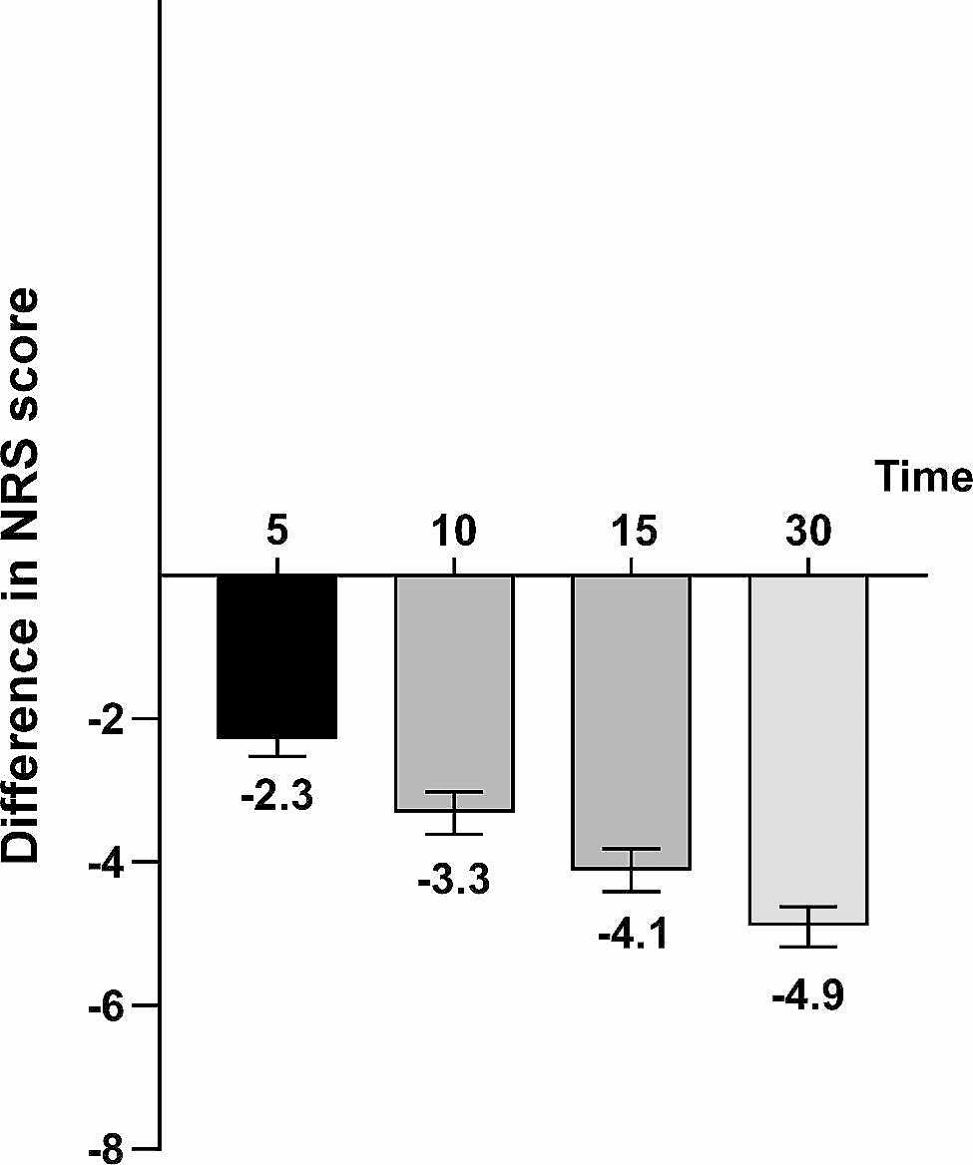
Effective pain relief from baseline to FBSF administration over time. Decline in NRS scores in cancer patients with BTcP episodes at 5, 10, 15, and 30 min after FBSF administration. Data are presented with mean and standard error of mean

Next, the decline in pain NRS score over time for each patient following FBSF administration was calculated during the onset of each BTcP episode (Fig. 3). FBSF effectively reduced the pain score by 2.3 points, 3.3 points, 4.1 points and 4.9 points at 5, 10, 15 and 30 min, respectively. The difference in NRS decline gradually increased over time. FBSF administration reduced the intensity of BTcP episode by 37.8%, 57.1%, 71.6%, and 84.3% at 5, 10, 15, and 30 min, respectively. In summary, use of the analgesic FBSF can rapidly and effectively reduces pain intensity of BTcP in cancer patients.

### Safety and satisfaction of FBSF analgesic for BTcP episodes in cancer patients

**Fig. 4:**
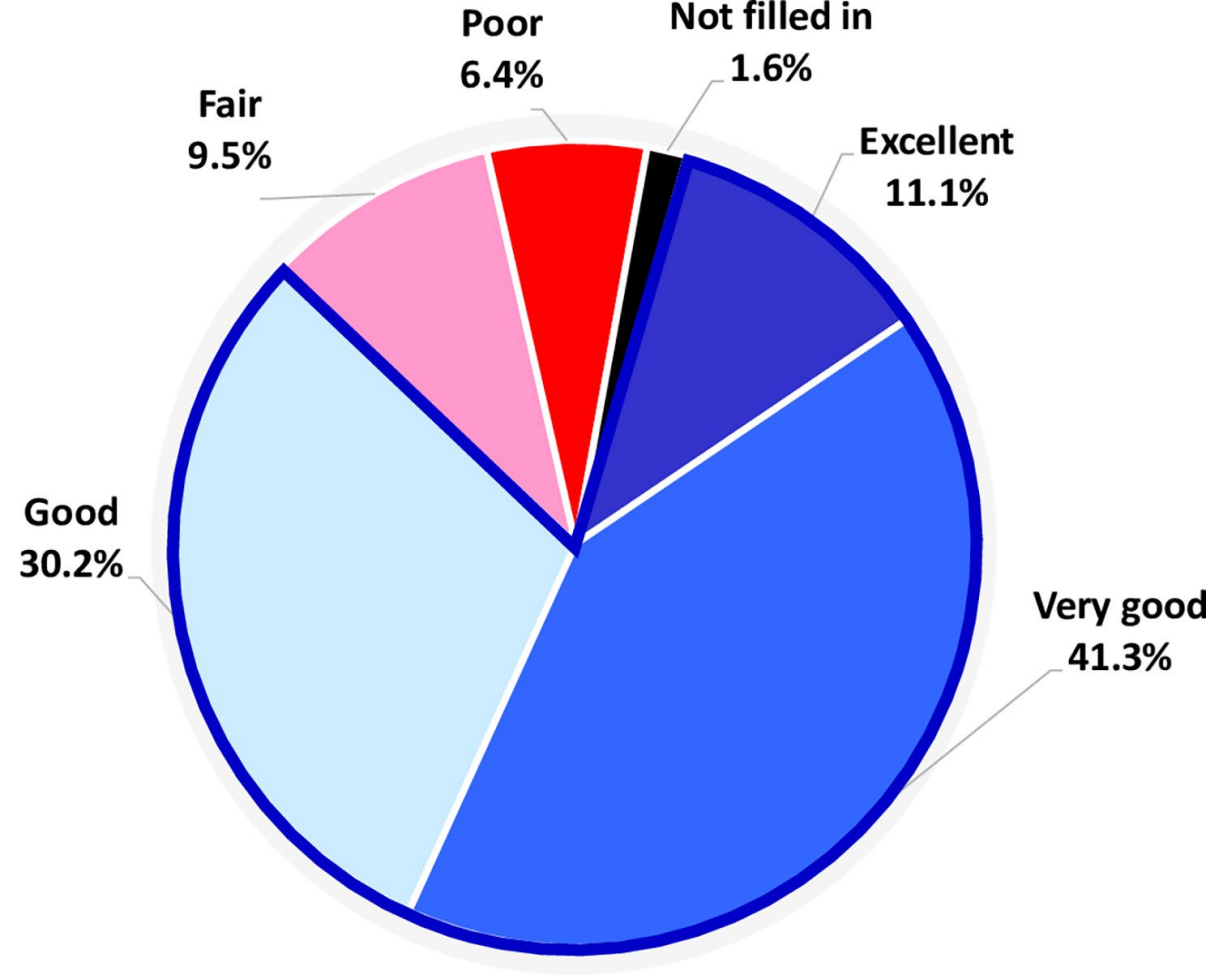
Pie-chart of overall satisfaction with FBSF in managing BTcP episodes

In the package insert of PBSF, serious adverse reactions and other potential safety hazards are listed to provide warning and precautions for prescribers and users. Throughout the study, AEs and SAEs were closely monitored. During the dose-finding and observation period, 2 of 30 patients reported FBSF relative adverse drug reaction following the use of analgesic FBSF for BTcP episodes. The side effects reported included somnolence (one patient), malaise (one patient), and dizziness (one patient), all of which were grade 1/2 adverse events. No respiratory depression was observed in any patient throughout the study period. Only one SAE was reported during the study. The SAE of septic shock was considered not related to FBSF treatment but to the progression of late-stage disease. All patients rated their satisfaction with FBSF for BTcP management. Figure 4 shows the percentage of satisfaction levels with the FBSF. Among the 63 episodes of BTcP, 11.1%, 41.3%, and 30.2% were rated as excellent, very good, and good satisfaction with FBSF. Conversely, only 4 (6.4%) BTcP episodes were rated as poor.

## Discussion

BTcP is a common clinical symptom that markedly and negatively impact patient’s survival outcome and health-related quality of life. The results of this study demonstrate that dose proportionality of FBSF based on opioid regimen for baseline pain management is effective and well tolerated. The only patient who failed to relieve pain with FBSF at the first episode of BTcP achieved meaningful pain relief after dose titration to 400 µg during the dose-finding period. Notably, self-administration of FBSF by cancer patients was effective in relieving meaningful pain intensity by nearly 50% within 5 min and 73% within 10 min due to the advantages of FBSF in formulation and pharmacokinetics. Within 10 min, FBSF can effectively reduce the pain score by 3.3 points, which is equivalent to reducing one pain level. The safety results reported here are similar to those of other FBSF studies, with only mild adverse events that resolved spontaneously. Greatly, more than 80% of cancer patients give favorable ratings to BTcP episodes treated with FBSF. For opioid-tolerant cancer patients, FBSF is recommended as first-line treatment to rapidly and effectively relieve BTcP.

In the first dose titration study of FBSF by Chiou et al. [23], FBSF was administrated in a dose titration manner until meaningful pain relief was achieved, and the dose was not allowed to be adjusted during the subsequent 7-day maintenance period even if the disease progressed. However, 4.6% BTcP episodes still required under the administration of FBSF, which prompted the development of dose proportionality studies of FBSF. In a pilot study exploring the FBSF in dose proportional to opioid regimen for background analgesia [22], the percentage of rescue medication was reduced to 2.2%. In addition, 75.8% of BTcP events were rated as satisfactory, which may benefit from the dose proportional strategy to reduce dose titration time and poor analgesia due to insufficient dose. It is speculated that the need for rescue medication due to inadequate analgesia may be largely attributable to the study design, which did not allow adjustment of ATC doses for background pain. In clinical practice, however, opioid dose adjustment for managing persistent pain is common in advanced cancer patients and is also recommended by National Comprehensive Cancer Network (NCCN) guideline [26, 27]. In order to be more in line with clinical practice as the intensity of background pain and BTcP may exacerbate over time, this study adopted the dose proportional strategy, and physicians were allowed to adjust the doses of ATC and FBSF during the 14-day observation period, depending on their clinical judgment and patient’s condition. In addition, patients self-assessed their pain intensity at very short intervals (5, 10, 15, and 30 min) after FBSF administration to better understand the rapid onset of FBSF. As a result, ATC dose escalation did occur in 12 (4.4%) cancer patients due to increased background pain, while FBSF dose escalation occurred in 2 (7.4%) patients during the 14-day observation period. On the other hand, there seems to be a growing consensus that the occurrence of BTcP episodes also be attributed to end-of dose failure, referring to the analgesia wearing off before the next usual medication [28, 29]. In this study, however, no BTcP episodes occurred at dosing interval of ATC opioid medication, that is, the opioid efficacy dropped below the analgesic level. In other words, all BTcP episodes in this study were caused by the tumor itself and/or the treatment received. In summary, well-controlled background cancer pain may minimize the occurrence of BTcP, while self-administration of fast-acting FBSF on demand can rapidly reduce pain intensity of BTcP adequately to maintain health-related quality of life to a great extent.

Notably, half of BTcP events were well-controlled within 5 min after FBSF administration, and nearly three-quarters of pain intensity of BTcP episodes were effectively relieved within 10 min. Furthermore, it need to emphasize that the definition of meaningful pain relief used in this study is stricter than in other studies: a meaningful pain relief was considered only when the final NRS score fell to 3 or less, not the 3-point or ≥ 33.3% reduction as defined in other studies. Therefore, the results of this study are gratifying for the rapid onset of BTcP with moderate-to-high pain intensity, and are not inferior or even superior to other studies using intranasal fentanyl spray (INFS), fentanyl buccal tablet (FBT), or OTFC [30–33]. In a multicentre crossover trial of BTcP [31], the proportions of ≥ 33.3% reduction in pain intensity after OTFC and INFS treatment were 6.8% and 25.3% at 5 min and 23.6% and 51.0% at 10 min, respectively. In two randomized controlled trials of FBT [32, 33], only about 13% of BTcP episodes achieved a ≥ 33% improvement in pain intensity 15 min after FBT administration. Future prospective trials with large sample size are warranted to compare the rapid analgesic effects of different clinically approved fentanyl formulations in BTcP episodes and patient satisfaction.

Since previous studies showed that OTFC treatment exhibited similar efficacy in both nociceptive and neuropathic pain [34], we surveyed and further categorized the types of background pain in our patients. Among the 30 patients, there were 3 cases (10.0%) of neuropathic cancer pain and 17 cases (56.7%) of nociceptive somatic pain. The remaining 10 cases (33.3%) had a mixed syndrome with other types of pain, including somatic pain, visceral pain, and neuropathic cancer pain [35]. Further analyses were attempted to explore the association between pain type and the efficacy FBSF for pain relief or the prevalence of BTcP, but the analyses could not be performed due to the small sample size of this study.

The study has several limitations, including the small sample size and lack of a control group. In addition, the study was conducted at a single center in Taiwan, which may limit its generalizability. However, the results were in line with findings of the previous study, with data further revealing the changes in pain intensity at several timepoints within 30 min of FBSF administration. Future prospective studies with large samples are needed to corroborate the findings of this study.

## Conclusions

Although not every cancer patient can be cured, every patient has their right to receive effective analgesics in the ethical point of view to eliminate or alleviate existing pain, especially for moderate-to-high pain intensity of BTcP episode. This study provides clinical evidence that FBSF can be administrated “on demand” by cancer patients at the onset of BTcP. Initial FBSF given in dose proportional to the background opioid regimen for controlling BTcP is effective and safe in clinical practice. On-demand self-administration of FBSF provides rapid-onset analgesia within 10 min to adequately reduce pain intensity in BTcP episodes. While a proportional method is employed to determine an effective starting dose for expediting the remission of the patient’s BTcP, the evolving condition of cancer patients necessitates adjusting the actual clinical dosage of ATC and FBSF based on the patient’s ongoing status.

### Electronic supplementary material

Below is the link to the electronic supplementary material.

Supplementary Material 1

Supplementary Material 2

## Data Availability

All data generated or analyzed during this study are included in this published article and its supplementary information files.
