# Supplementary material for: Effectiveness of fentanyl buccal soluble film in cancer patients with inadequate breakthrough pain control
Source: BMC Palliat Care. 2024 Jun 14;23:150. doi: 10.1186/s12904-024-01483-7 (PMC11177451; doi:10.1186/s12904-024-01483-7)
Supplement: Supplementary file 1 — Supplementary Material 1 [file 12904_2024_1483_MOESM1_ESM.docx]

**Supplementary Table 1. Conversion of FBSF doses based on oral opioid dose used in patients during the 7-day treatment period (N=30)**

| ATC dose |  | ROO dose | | | |  | Patients (%) | |
| --- | --- | --- | --- | --- | --- | --- | --- | --- |
| Oral morphine (mg) |  | FBSF (μg) | | | |  |  |  |
|  |  | 200 | 400 | 600 | 800 |  |  |  |
| 60-119 |  | 18 |  |  |  |  | 18 | (60.0%) |
| 120 – 179 |  | 3 | 5 |  |  |  | 8 | (26.7%) |
| 180 – 239 |  |  |  | 3 |  |  | 3 | (10.0%) |
| 240+ |  |  |  |  | 1 |  | 1 | (3.3%) |
| Patients (%) |  | 21 (70%) | 5 (16.7%) | 3 (10.0%) | 1 (3.3%) |  |  |  |

Abbreviation: FBSF, fentanyl buccal soluble film; ATC, around-the-clock; BTcP, breakthrough cancer pain, ROO, rapid-onset opioid.

**Supplementary Table 2. Conversion of FBSF dose from current regimen of opioid**

| ATC dose | | | BTP dose |
| --- | --- | --- | --- |
| Morphine  (mg/day) | | Transdermal fentanyl | Painkyl |
| IV / SC | Oral | μg/hour | μg/time |
| 20 | 60 | 25 | 200 |
| 40 | 120 | 50 | 400 |
| 60 | 180 | 75 | 600 |
| 80 | 240 | 100 | 800 |
| 100 | 300 | 125 | 1000 |
| 120 | 360 | 150 | 1200 |

Abbreviations: IV, intravenous; SC, subcutaneous.
