# Supplementary material for: Effectiveness of fentanyl buccal soluble film in cancer patients with inadequate breakthrough pain control
Source: BMC Palliat Care. 2024 Jun 14;23:150. doi: 10.1186/s12904-024-01483-7 (PMC11177451; doi:10.1186/s12904-024-01483-7)
Supplement: Supplementary file 2 — Supplementary Material 2 [file 12904_2024_1483_MOESM2_ESM.pdf]

Treatment Satisfaction Questionnaire for Medication (Version 4.0)

Pain and Satisfaction Survey Questionnaire

Hospital/Subject Code: _______________ / ______________
Date of Painkyl usage: _____ /_____ /______

Instructions: Please take some time to think about your level of pain after the use of Painkyl for relieving the breakthrough cancer pain (BTcP), as well as the satisfaction or dissatisfaction with the medication you are taking in this clinical trial. For each question, please place a single check mark next to the response that most closely corresponds to your own experiences.

Please have the patient fill out the pain score assessment

Before medication (BTcP onset)

What was your level of pain before using Painkyl at the onset of BTcP? (0 indicates no pain, and 10 indicates extremely painful)

☐1 ☐2 ☐3 ☐4 ☐5 ☐6 ☐7 ☐8 ☐9 ☐10

Five minutes after using Painkyl for relieving BTcP

What is your level of pain 5 minutes after using Painkyl? (0 indicates no pain, and 10 indicates extremely painful)

☐1 ☐2 ☐3 ☐4 ☐5 ☐6 ☐7 ☐8 ☐9 ☐10

Ten minutes after using Painkyl for relieving BTcP

What is your level of pain 10 minutes after using Painkyl? (0 indicates no pain, and 10 indicates extremely painful)

☐1 ☐2 ☐3 ☐4 ☐5 ☐6 ☐7 ☐8 ☐9 ☐10

Fifteen minutes after using Painkyl for relieving BTcP

What is your level of pain 15 minutes after using Painkyl? (0 indicates no pain, and 10 indicates extremely painful)

☐1 ☐2 ☐3 ☐4 ☐5 ☐6 ☐7 ☐8 ☐9 ☐10

Thirty minutes after using Painkyl for relieving BTcP

What is your level of pain 30 minutes after using Painkyl? (0 indicates no pain, and 10 indicates extremely painful)

☐1 ☐2 ☐3 ☐4 ☐5 ☐6 ☐7 ☐8 ☐9 ☐10

Please have the patient fill out the level of satisfaction

Thirty minutes after using Painkyl, what is your satisfaction level with this pain treatment.

- ☐1 Poor
- ☐2 Fair
- ☐3 Good
- ☐4 Very Good
- ☐5 Excellent

Please have healthcare personnel fill out the pain medication usage status

Did the patient use any other rescue medication at this onset of BTcP?

- ☐1 Yes
- ☐2 No

Did the patient use an adjusted dose of Painkyl for this onset of BTcP?

- ☐1 Yes
- ☐2 No
